# Uncertainty of treatment efficacy moderates placebo effects on reinforcement learning

**DOI:** 10.1038/s41598-024-64240-z

**Published:** 2024-06-22

**Authors:** Nick Augustat, Dominik Endres, Erik M. Mueller

**Affiliations:** Department of Psychology, University of Marburg, Marburg, Germany

**Keywords:** Placebo effect, Reward, Psychology, Human behaviour

## Abstract

The placebo-reward hypothesis postulates that positive effects of treatment expectations on health (i.e., placebo effects) and reward processing share common neural underpinnings. Moreover, experiments in humans and animals indicate that reward uncertainty increases striatal dopamine, which is presumably involved in placebo responses and reward learning. Therefore, treatment uncertainty analogously to reward uncertainty may affect updating from rewards after placebo treatment. Here, we address whether different degrees of uncertainty regarding the efficacy of a sham treatment affect reward sensitivity. In an online between-subjects experiment with N = 141 participants, we systematically varied the provided efficacy instructions before participants first received a sham treatment that consisted of listening to binaural beats and then performed a probabilistic reinforcement learning task. We fitted a Q-learning model including two different learning rates for positive (gain) and negative (loss) reward prediction errors and an inverse gain parameter to behavioral decision data in the reinforcement learning task. Our results yielded an inverted-U-relationship between provided treatment efficacy probability and learning rates for gain, such that higher levels of treatment uncertainty, rather than of expected net efficacy, affect presumably dopamine-related reward learning. These findings support the placebo-reward hypothesis and suggest harnessing uncertainty in placebo treatment for recovering reward learning capabilities.

## Introduction

Our expectations shape how we remember the past^1,2^, experience the present^3–5^ and predict the future^6^. Expectations are built on prior experience and are strongly influenced by unexpected events^7^. Unexpected events create *prediction errors*, defined as positive or negative deviations from expectations, and drive learning^8–10^. The extent, to which single prediction errors influence expectations, is called the *learning rate*.

A number of psychological phenomena are subject to differences in the individual weighting of reward prediction errors^11,12^, which may be altered through placebo interventions^13,14^. A placebo intervention releases no medical agent into the organism, nevertheless, inducing positive treatment expectations towards a sham or open-label placebo trial may prove beneficial to several health outcomes, such as pain or depression^15,16^. It is thus of great interest for novel therapeutic approaches to disentangle and harness the supplementary factors of placebos contributing to symptoms improvement.

Neurobiological studies on placebo effects in pain and depression suggest that placebo effects involve dopamine-related processes^17^. Specifically, the placebo-reward-hypothesis^18^ states that the effect of positive treatment expectations may directly be linked to striatal dopamine release, which in turn innervates the reward circuitry and thereby may mitigate symptoms of pain and depression. More specifically, it proposes that placebos rely on similar or identical neural underpinnings as rewards, and that the nucleus accumbens in the striatum constitutes the locus of dopamine-related action in response to reward expectations in anticipation of therapeutic improvement. This implies a particularly useful role of placebos in the treatment of mental disorders associated with attenuated processing of and learning from rewards, as for instance major depressive disorder^19^.

Reward sensitivity may be increased by inducing positive treatment expectations. Particular evidence stems from a study on Parkinson’s disease^20^, although a study on healthy participants provided mixed findings^21^. In a prior study, we could find that learning rates for gain increased through a positive treatment expectation intervention in healthy participants^22^. Before the participants performed a probabilistic reinforcement learning task, they were told that they either received an inert substance or an antidepressant pill, but, actually, all participants received an inert substance pill. However, the highest learning rates for gain were observed in the antidepressant expectation group. If this expectation-induced increase of learning rates accounts for the expectation-induced increase in therapeutic efficacy commonly observed in antidepressant trials^23–25^, it could be hypothesized that *learning rates for gain increase with higher subjective probabilities of receiving an active antidepressant substance*, potentially leading to increased symptoms improvement.

In this regard, the benefits of the placebo effect would be presumed to follow a monotonic pattern, such that high (vs. low) expectations towards a beneficial effect would result in increased learning rates for gain (*monotonicity hypothesis*). Nevertheless, additional factors may have to be taken into account: theories define uncertainty as a part of expectations^26,27^ with clinical implications^28^. Given the placebo effect primarily relies on expectations, uncertainty regarding the probability of treatment efficacy could constitute an additional feature of placebo effects.

Similar to the link between placebo and reward, uncertainty as well makes a significant contribution to reward sensitivity^26,29^. Accordingly, in an unknown or variable environment, an ideal observer should update its expectations more from recent observations than in a known and static environment. In this regard, previous studies have shown maximum tonic firing of striatal dopaminergic neurons during *maximum reward uncertainty*^30,31^, i.e., a 50 percent probability of reward delivery. Similar effects have been observed regarding dopamine release^32–34^, even though tonic firing may not necessarily affect dopamine release^35^. This tonic enhancement is observed in classically as well as operantly conditioned responses and presumed to reflect state uncertainty, although its specific role is not fully understood yet^36^. Given reward uncertainty increases tonic striatal dopaminergic firing, which in turn might facilitate quick updating from rewards, *learning rates for gain should reach its peak under maximum reward uncertainty* (*uncertainty hypothesis*). Consistent with the uncertainty hypothesis (rather than the monotonicity hypothesis), a previous study^37^ addressed the effect of placebo expectancy on learning rates for gain under high vs. low uncertainty regarding treatment efficacy and found increased learning rates for gain in a high (vs. low) uncertainty condition. Therefore, when varying the expected treatment efficacy systematically, the pattern of the learning rates for gain expected under this hypothesis would exhibit an *inverted-U-shape*, that is, a peak at maximum treatment uncertainty with monotonic increase on the left-hand side towards the peak, and monotonic decrease on the right-hand side towards the largest expected treatment efficacy.

Computational models within the reinforcement learning framework not only allow estimating effects of uncertainty on learning rates^3,38,39^, but have also been proven useful for investigating aberrant processes in mood disorders^40,41^. A mechanistic approach includes the computation of learning rates to capture the mapping of reward prediction errors onto future reward expectations. A higher learning rate reflects a stronger weighting of recent outcomes, which would be highly useful in a volatile environment, such as everyday life. In turn, a low value indicates a stronger integration of cumulative experiences serving best in a stable environment^28,38^. Separate learning rates for gain and loss capture how differential sensitivity towards positive and negative feedback influences the expected choice value, resembling positive and negative reward prediction errors. In context of mood and anxiety disorders, learning rates have been assumed to exhibit a shift towards altered updating from negative feedback^40^.

In the present study, we asked if the learning rate is related to treatment uncertainty such that the relationship between increasing treatment efficacy probabilities and learning rate follows an inverted-U pattern similar to the tonic dopamine pattern exhibited under different reward probabilities^30,32^. If so, individuals receiving a placebo treatment under different efficacy probabilities, analogously to varying reward probabilities, would exhibit increased sensitivity to rewards particularly under intermediate treatment efficacy probabilities where uncertainty is maximal. Alternatively, a monotonic increase of learning rate as a function of treatment efficacy probability would be consistent with recommendations from clinical findings to maximize treatment certainty for antidepressant trials^24^.

**Figure 1:**
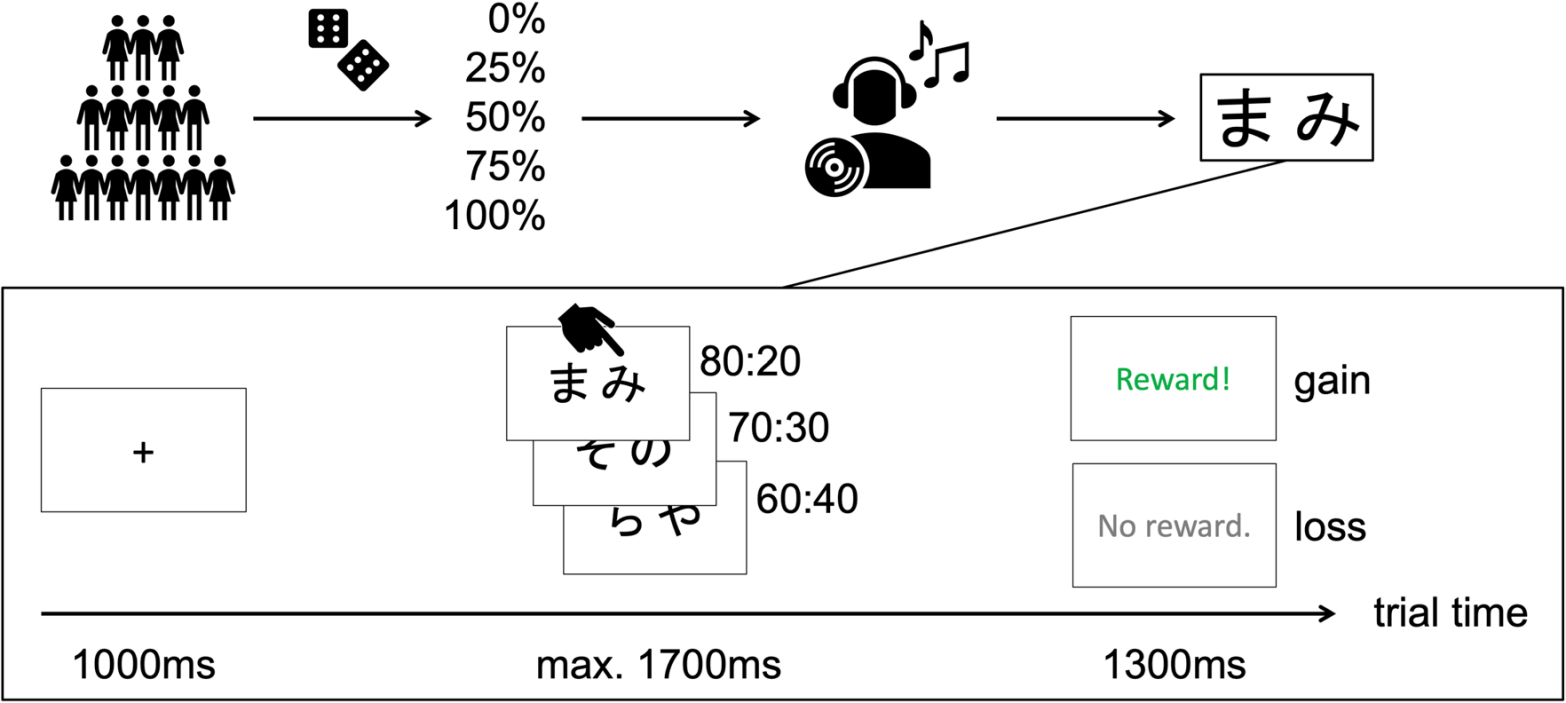
Experimental design and probabilistic RL task design. Top, Participants were randomly assigned into one of five groups differing in verbally provided probabilistic treatment efficacies (i.e., expected treatment efficacies) before listening to binaural beats as sham treatment. Afterwards, a probabilistic reinforcement learning task was performed. Bottom, The task constituted of a fixation period followed by stimulus presentation with a time limit for choice-making and a subsequent probabilistic feedback. The fixed reward probabilities of each pair were 80% to 20%, 70% to 30%, and 60% to 40% for the more vs. less frequently rewarded stimulus, respectively. Crucially, although the reward probabilities indicate that one stimulus is, on average, preferable over the other stimulus, choosing those stimuli exhibiting lower reward probabilities has been rewarded at times.

In order to test the *monotonicity hypothesis* against the *uncertainty hypothesis*, we collected behavioral data online and randomly assigned participants into five groups differing with regard to the *expected treatment efficacy*, i.e., the purported probability of treatment efficacy, from 0 to 100 percent in steps of 25 percent (Fig. 1, top; see Methods for details). Participants underwent a sham auditive treatment using binaural beats before indicating their treatment expectations. Binaural beats are two sinusoidal audio waveforms with different frequencies played separately on each ear and evoke an illusory difference tone equal to the frequency difference of the waveforms, which is perceived only by the listening individual. In public media, binaural beats are often promoted as a cognitive enhancement procedure, in spite of no substantial empirical evidence^42^. Therefore, harnessing this pseudo-treatment promised efficient use as inactive placebo treatment in an online approach. The participants subsequently performed a well-established probabilistic reinforcement learning task that has been shown to be sensitive to striatal dopamine^43^. In this task participants are simultaneously presented three pairs of two distinct stimuli each with different reward probabilities (80:20, 70:30 and 60:40, respectively) and participants have to find out by trial-and-error, which stimulus is more often rewarded. We applied a Q-learning algorithm^37,44^ with separate learning rates for both gain and loss trials to the choice data.

## Results

### Expected treatment efficacy did not affect behavioral task performance

**Figure 2:**
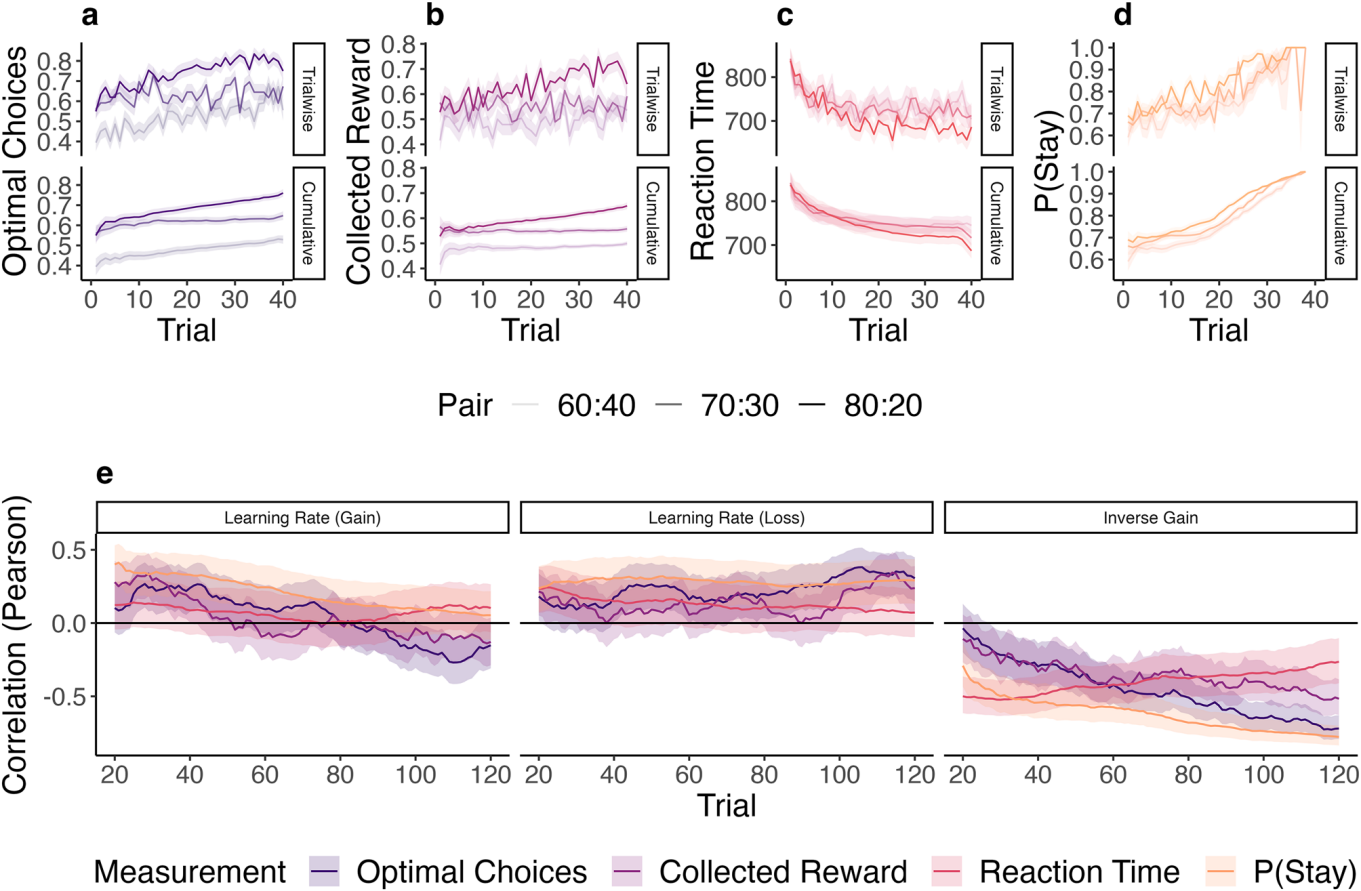
Behavioral measurements over the course of the probabilistic RL task and their correlation with individual RL parameter posterior means. (**a**, **b**) The development of reinforcement contingency is shown within each stimulus pair for optimal choices and for collected reward, respectively. For each trial, response values (0 or 1; top) and individual cumulative means (mean of all response values from trial 1 to the depicted trial number; bottom) were averaged across all participants. With increasing trial number, participants learned to make more optimal choices, particularly for the 80:20-pair. Shaded area indicates the standard error at each trial. The pair is designated by opacity of the lines. (**c, d**) The same procedure as in (**a**) and (**b**) was applied to reaction times (**c**), indicating faster choices with increasing trial number, and the stay probability (**d**), i.e., probability of three equal consecutive choices given two equal preceding choices. (**e**) Pearson’s product-moment correlations between RL parameters and individual means for count of optimal choices (purple), collected reward (magenta), reaction time (red), and stay probability (orange). RL parameters are represented on the x-axis, Pearson’s product-moment correlation coefficients on the y-axis. To obtain the temporal correlations, a sliding window of 20 trials was moved in steps of 1, where the first trial window (at trial 20) consisted of trials 1 to 20. Shaded areas represent the uncorrected 95% confidence interval at for each trial window.

Before assessing computational differences in task performance, we analyzed four aggregate measurements of behavioral performance typically encountered in behavioral experiments, i.e., reaction time (time between stimulus presentation and choice), optimal choices (task average of accuracy, or selecting the more rewarding stimulus under initially unknown reward probabilities) collected reward (task average of positive feedback), and stay probability (the probability of three equal consecutive choices given two preceding equal choices). Figure 2 (top) shows trial-wise aggregates over the course of the task. A hierarchical generalized mixed-effects regression with log-link function including the provided probability of treatment efficacy as fixed effect and participant ID as random effect was tested against an intercept-only model. The findings revealed evidence for indifference regarding individual reaction times (*χ*^2^(4) = 3.04, *p* = 0.551, BF_10_ < 0.001), the count of optimal choices (*χ*^2^(4) = 1.64, *p* = 0.802, BF_10_ < 0.001), collected reward (*χ*^2^(4) = 3.99, *p* = 0.407, BF_10_ < 0.001), and stay probability (*χ*^2^(4) = 1.94, *p* = 0.746, BF_10_ < 0.001). Therefore, expected treatment efficacy did not show effects on conventional behavioral measurements of overall task performance.

### Expected treatment efficacy alters reinforcement learning

**Figure 3:**
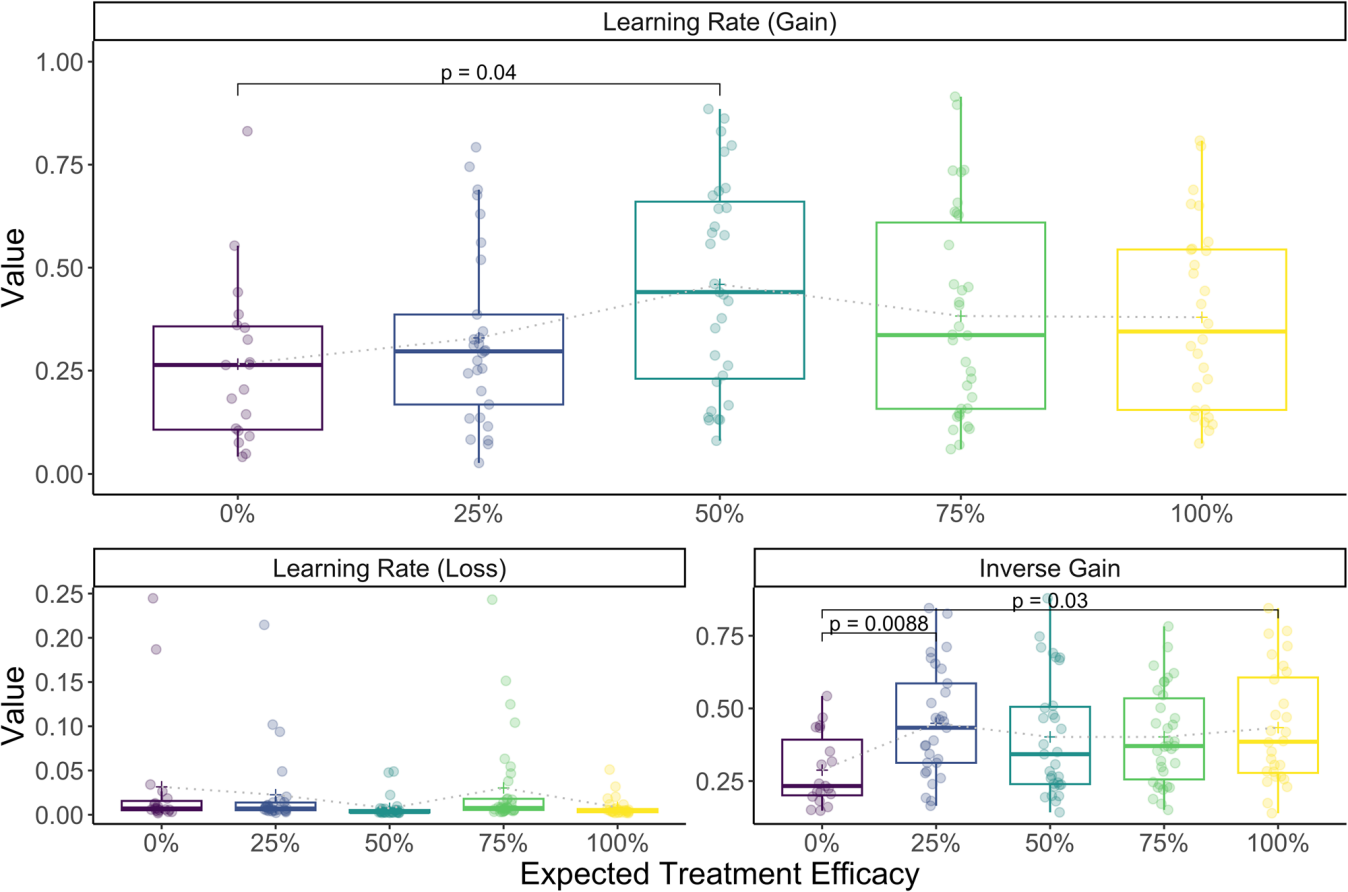
Individual RL parameters separately for each treatment group. Treatment groups are represented on the x-axis, and transformed (constrained) individual parameter posterior means are shown on the y-axis. Box plots represent the group-level median (thick horizontal bar), quartiles and whiskers (1.5*IQR). Group-level means are depicted as a cross and connected with a thin dotted line. P-values are displayed, if Bonferroni-correction for tenfold comparisons was significant at *p* < .05.

**Figure 4:**
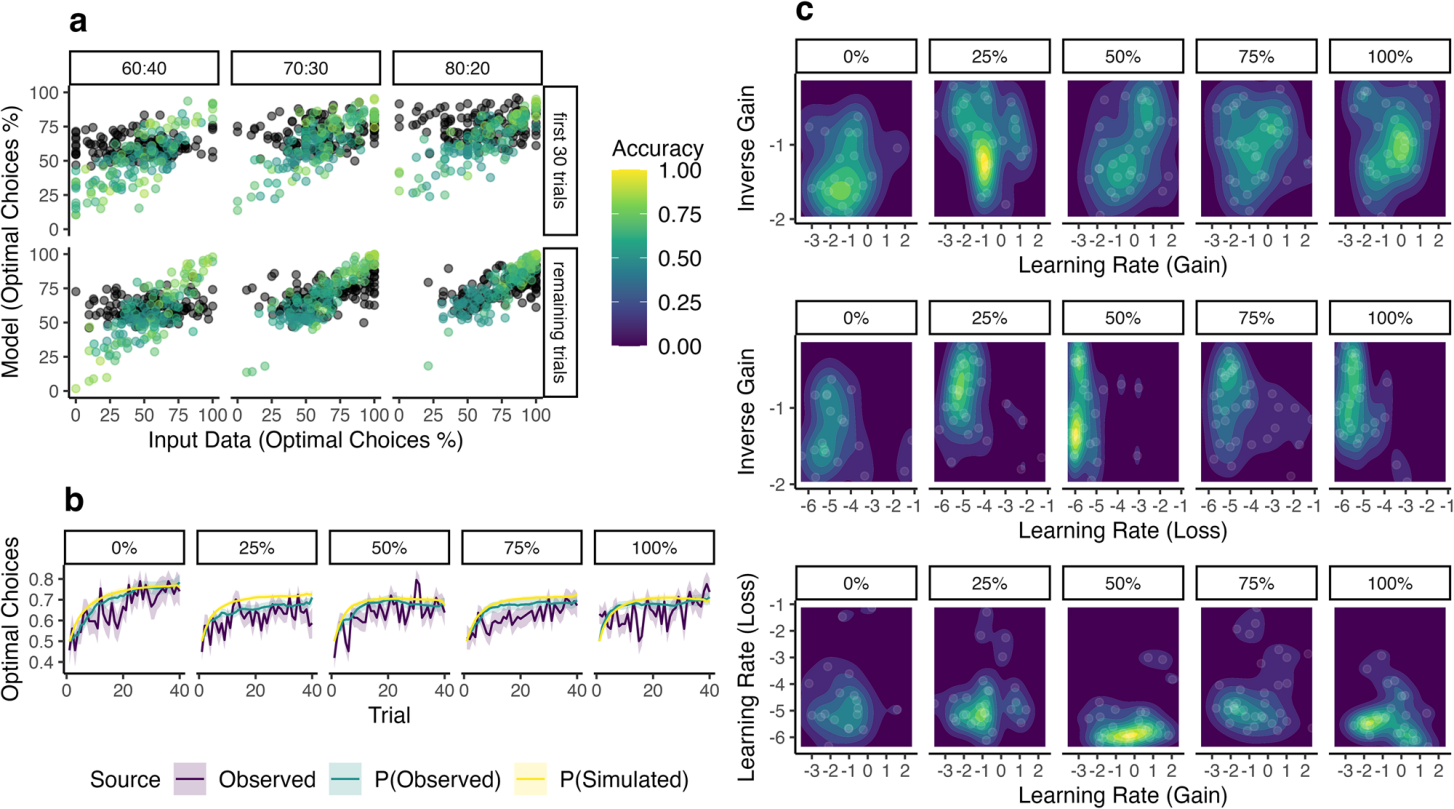
Model recovery. (**a**) Observed means of participants’ optimal choices (x-axis) contrasted against means of optimal choices using the obtained RL parameters on simulated task data (black dots) and sampled from posterior likelihoods of observed choices (colored dots), separately for each participant’s first 30 trials (top row) and all remaining trials (bottom row). Brighter colors indicate a higher model accuracy for the observed choices in terms of a higher mean posterior likelihood for observed choices. (**b**) Trial-wise means for observed optimal choices (violet), model likelihood of optimal choices on observed data (green), and model likelihood of optimal choices on simulated data (yellow), averaged across all pairs within subjects first, and averaged within groups afterwards. The x-axis denotes the trial number within a pair (max. 40). (**c**) Pairwise density estimates of individual RL parameter posterior means on the unconstrained scale used for sampling. Note that the color levels are relative to the data depicted within each row, not across all rows. Brighter colors indicate higher density.

Next, we investigated group differences concerning RL parameters. As previously noted, learning rates reflect the strength as to which novel feedback updates the expected outcome value (here: of a stimulus), separately for positive (gain) and negative (loss) feedback, whereas the inverse gain parameter modulates the choice probability, i.e., how “rational” a learner integrates expected value differences in its decisions. We performed Markov chain Monte Carlo (MCMC) sampling for parameter estimation on individual decision data and took the individual posterior means of RL parameters as a marker for individual reward sensitivity (see Methods for details; see Supplementary Fig. S1 for the posterior sampling distributions; Supplementary Fig. S2 for MCMC traces; and Supplementary Fig. S3 for posterior predictive distributions). To test the model assumption of separate updating from gain and loss, we performed a generalized mixed model with participant ID as random effect, which indicated that preceding reward feedback significantly interacted on the subsequent stay probability within stimuli pairs supporting the assumptions of outcomes to differently drive subsequent choices (*χ*^2^(7) = 5.75, *p* = 0.016, BF_10_ < 0.171). In other words, different reward feedback in two consecutive equal choices significantly decreased the stay probability in the subsequent choice. Further, our model showed reasonable fit to the data (63.7 percent model accuracy in terms of the mean likelihood of observed choices; see Methods for details), and no group differences could be observed with regard to the fit (*F*(4,136) = 0.39, *p* = 0.814, BF_10_ = 0.042). Three one-way ANOVAs were computed with the provided probability of treatment efficacy grouping factor as fixed between-subjects effect and each RL parameter on its original, normally-distributed unconstrained scale used for sampling (logit-scale for learning rates to ensure transformed values between 0 and 1, and log-scale for inverse gain to set a lower limit of zero) as the dependent variable. The ANOVAs yielded significant group effects regarding learning rates for gain (*F*(4,136) = 2.47, *p* = 0.048, *η*^*2*^ = 0.07, 95% CI_*η2*_ [0.00, 1.00], *ω*^*2*^ = 0.04, 95% CI_*ω2*_ [0.00, 1.00], BF_10_ = 0.77), learning rates for loss (*F*(4,136) = 5.84, *p* < 0.001, *η*^*2*^ = 0.15, 95% CI_*η2*_ [0.05, 1.00], *ω*^*2*^ = 0.12, 95% CI_*ω2*_ [0.03, 1.00], BF_10_ = 126) as well as the inverse gain parameter (*F*(4,136) = 2.96, *p* = 0.022, *η*^*2*^ = 0.08, 95% CI_*η2*_ [0.01, 1.00], *ω*^*2*^ = 0.05, 95% CI_*ω2*_ [0.00, 1.00], BF_10_ = 1.33). See Fig. 3 for transformed individual posterior means by group and significant post-hoc comparisons. Model and parameter recovery are shown in Fig. 4. Pairwise post-hoc comparisons are shown in Table 1.

**Table 1:** Post-hoc comparisons for individual RL parameter posterior means.

Parameter	Contrast	*d*	*SE*	*df*	CI
Learning rate (gain)	(0–25%)	0.06	0.30	136	[− 0.52, 0.64]
	(0–50%)	0.86	0.30	136	[0.27, 1.44]
	(0–75%)	− 0.16	0.29	136	[− 0.73, 0.4]
	(0–100%)	0.63	0.30	136	[0.04, 1.22]
	(25–50%)	0.80	0.26	136	[0.28, 1.32]
	(25–75%)	− 0.22	0.25	136	[− 0.72, 0.28]
	(25–100%)	0.57	0.27	136	[0.04, 1.1]
	(50–75%)	− 1.02	0.26	136	[− 1.53, − 0.51]
	(50–100%)	− 0.23	0.26	136	[− 0.74, 0.29]
	(75–100%)	0.79	0.26	136	[0.28, 1.31]
Learning rate (loss)	0–25%	0.06	0.30	136	[− 0.52, 0.64]
	0–50%	0.86	0.30	136	[0.27, 1.44]
	0–75%	− 0.16	0.29	136	[− 0.73, 0.4]
	0–100%	0.63	0.30	136	[0.04, 1.22]
	25–50%	0.80	0.26	136	[0.28, 1.32]
	25–75%	− 0.22	0.25	136	[− 0.72, 0.28]
	25–100%	0.57	0.27	136	[0.04, 1.1]
	50–75%	− 1.02	0.26	136	[− 1.53, − 0.51]
	50–100%	− 0.23	0.26	136	[− 0.74, 0.29]
	75–100%	0.79	0.26	136	[0.28, 1.31]
Inverse gain	0–25%	0.06	0.30	136	[− 0.52, 0.64]
	0–50%	0.86	0.30	136	[0.27, 1.44]
	0–75%	− 0.16	0.29	136	[− 0.73, 0.4]
	0–100%	0.63	0.30	136	[0.04, 1.22]
	25–50%	0.80	0.26	136	[0.28, 1.32]
	25–75%	− 0.22	0.25	136	[− 0.72, 0.28]
	25–100%	0.57	0.27	136	[0.04, 1.1]
	50–75%	− 1.02	0.26	136	[− 1.53, − 0.51]
	50–100%	− 0.23	0.26	136	[− 0.74, 0.29]
	75–100%	0.79	0.26	136	[0.28, 1.31]

For each contrast, Cohen’s d (*d*) is shown with standard error (*SE*), degrees of freedom (*df*) and the 95% confidence interval (CI).

### Learning rates for gain exhibit an inverted-U-shape for expected treatment efficacy

In order to investigate the shape of the group effect according to our hypotheses and whether it exhibits a monotonic pattern vs. inverted-U-shape, we tested a positive monotonic vs. inverted-U-shaped relationship with regard to learning rates for gain as index of reward sensitivity. For this purpose, the number of MCMC samples, in which the group-level estimates matched a (1) monotonic or (2) an inverted-U-shaped pattern, was counted and divided by the number of samples collected. We took all 80,000 MCMC group-level posterior samples for the learning rates for gain (*α*_gain_), and computed the percentage of samples exhibiting either pattern on group-level estimates (*μ*). We assumed *μ*_*α*,gain_(0%) < * μ*_*α*,gain_ (25%) < *μ*_*α*,gain_ (50%) < *μ*_*α*,gain_ (75%) < *μ*_*α*,gain_ (100%) for a monotonic relationship, and *μ*_*α*,gain_ (0%) < *μ*_*α*,gain_(25%) < *μ*_*α*,gain_ (50%) > *μ*_*α*,gain_(75%) > *μ*_*α*,gain_(100%) for an inverted-U-shaped relationship. A monotonic increase was observed in 2.1 percent of the samples, whereas an inverted-U-shaped pattern was present in 17.4 percent of the MCMC samples. By omitting the 25% (*unlikely*) and 75% (*likely*) conditions, the percentage increased to 22.8 percent for the monotonical increase, and 68.6 percent for the inverted-U-pattern. All pairwise comparisons of the group-level posterior means are shown in Table 2.

**Table 2:** Pairwise post-hoc comparisons of group-level parameter posterior samples.

Parameter	Contrast	Mean	CI	pd (%)	ps	% in ROPE
Learning rate (gain)	0–25%	− 0.30	[− 1.78, 1.13]	65.75	0.61	10.42
	0–50%	− 0.97	[− 2.37, 0.42]	91.34	0.89	4.71
	0–75%	− 0.59	[− 1.98, 0.78]	80.15	0.76	8.29
	0–100%	− 0.57	[− 1.94, 0.84]	79.10	0.75	8.41
	25–50%	− 0.67	[− 2.07, 0.72]	82.63	0.79	7.65
	25–75%	− 0.30	[− 1.66, 1.04]	66.42	0.61	11.07
	25–100%	− 0.28	[− 1.66, 1.09]	65.34	0.60	11.19
	50–75%	0.38	[− 0.91, 1.68]	71.34	0.66	11.00
	50–100%	0.39	[− 0.97, 1.68]	71.89	0.67	10.75
	75–100%	0.02	[− 1.27, 1.32]	51.13	0.45	12.98
Learning rate (loss)	0–25%	0.04	[− 1.96, 1.96]	51.42	0.47	8.80
	0–50%	0.90	[− 1.71, 3.82]	74.59	0.71	6.60
	0–75%	− 0.20	[− 1.98, 1.51]	58.38	0.54	9.64
	0–100%	0.65	[− 1.65, 2.99]	70.87	0.67	7.11
	25–50%	0.86	[− 1.90, 3.98]	72.55	0.69	6.38
	25–75%	− 0.24	[− 2.04, 1.52]	59.90	0.55	9.36
	25–100%	0.61	[− 1.71, 3.06]	68.85	0.65	6.89
	50–75%	− 1.10	[− 4.06, 1.31]	81.46	0.79	6.07
	50–100%	− 0.25	[− 3.70, 3.12]	54.48	0.51	6.66
	75–100%	0.86	[− 1.24, 3.17]	78.18	0.75	6.51
Inverse gain	0–25%	− 0.43	[− 0.86, 0.00]	97.67	0.94	4.09
	0–50%	− 0.29	[− 0.69, 0.12]	92.27	0.82	15.58
	0–75%	− 0.32	[− 0.73, 0.08]	94.26	0.86	12.04
	0–100%	− 0.39	[− 0.80, 0.04]	96.44	0.91	6.88
	25–50%	0.14	[− 0.23, 0.51]	76.87	0.58	33.98
	25–75%	0.10	[− 0.27, 0.47]	71.19	0.51	37.36
	25–100%	0.04	[− 0.34, 0.42]	58.84	0.38	40.88
	50–75%	− 0.03	[− 0.37, 0.32]	57.53	0.35	44.88
	50–100%	− 0.09	[− 0.45, 0.27]	69.72	0.48	39.23
	75–100%	− 0.06	[− 0.42, 0.31]	62.85	0.42	41.34

*pd* is the probability of direction, *ps* is practical significance. Region of practical equivalence (ROPE) was set to [− 0.1, 0.1]. CI denotes the 95% confidence interval.

Further, we performed a hierarchical regression for the individual RL parameter posterior means comparing a linear-only with a combined linear and quadratic (as a special case of an inverted-U-shape) effect of numerical expected treatment efficacy. The expected treatment efficacy group factor (0–100% in steps of 25%) was transformed to centered numerical values ranging between 50 and − 50 with a mean of 0. Bayes factors are reported for comparisons against intercept-only models. The first model included a linear group term (*R*^2^ = 0.02, *F*(1,139) = 3.08, *p* = 0.081, BF_10_ = 0.73). A quadratic term was added to the second model (*R*^2^ = 0.05, *F*(2,138) = 3.72, *p* = 0.027, BF_10_ = 1.26; ∆*R*^2^ = 0.03, *F*(1,138) = 4.28, *p* = 0.040) providing support for the uncertainty hypothesis. At the same time, adding a quadratic term also revealed an additional significant linear effect of expected treatment efficacy in support of the monotonicity hypothesis, which increased from *b* = 0.005 to *b* = 0.006 (*p* = 0.046). For complete results, see Table 3.

**Table 3:** Regression results using learning rate for gain as the criterion.

Predictor	*b*	*b* 95% CI [LL, UL]	*beta*	*beta* 95% CI [LL, UL]	*sr*^*2*^	*sr*^*2*^ 95% CI [LL, UL]	*r*	Fit	Difference
(Intercept)	− 0.69**	[− 0.90, − 0.49]							
group	0.01	[− 0.00, 0.01]	0.15	[− 0.02, 0.31]	.02	[.00, .09]	.15		
								*R*^*2*^ = .022	
								95% CI[.00,.09]	
(Intercept)	− 0.46**	[− 0.76, − 0.16]							
group	0.01*	[0.00, 0.01]	0.17	[0.00, 0.33]	.03	[− .03, .08]	.15		
group_sq	− 0.00*	[− 0.00, − 0.00]	− 0.17	[− 0.34, − 0.01]	.03	[− .02, .08]	− .15		
								*R*^*2*^ = .051*	Δ*R*^*2*^ = .029*
								95% CI[.00,.13]	95% CI[− .02, .08]

A significant *b*-weight indicates the beta-weight and semi-partial correlation are also significant. *b* represents unstandardized regression weights. *beta* indicates the standardized regression weights. *sr*^*2*^ represents the semi-partial correlation squared. *r* represents the zero-order correlation. *LL* and *UL* indicate the lower and upper limits of a confidence interval, respectively.

*Indicates *p* < .05. ** indicates *p* < .01.

### Correlations of RL parameters and behavioral performance depend on task stage

Across the task, learning rate for gain did not significantly correlate with behavioral measurements, i.e., optimal choices (*r* = 0.02, 95% CI [− 0.14, 0.19]), collected reward (*r* = 0.04, 95% CI [− 0.13, 0.20]) and reaction time (*r* = 0.09, 95% CI [− 0.08, 0.25]), and stay probability (*r* = 0.05, 95% CI[− 0.11, 0.21]). Learning rate for loss correlated significantly with optimal choices (*r* = 0.31, 95% CI [0.16, 0.45]), collected reward (*r* = 0.25, 95% CI [0.09, 0.40]) and stay probability (*r* = 0.29, 95% CI [0.13, 0.44]), but not with reaction time (*r* = 0.15, 95% CI [− 0.01, 0.31]). The inverse gain parameter correlated numerically the strongest of all parameters with all behavioral measurements, i.e., count of optimal choices (*r* = − 0.62, 95% CI [− 0.72, − 0.51]), collected reward (*r* = − 0.64, 95% CI [− 0.73, − 0.53]), and reaction time (*r* = − 0.45, 95% CI [− 0.57, − 0.31]), and stay probability (*r* = − 0.77, 95% CI [− 0.83, − 0.70]). The higher the inverse gain parameter was, the lower were the count of optimal choices and collected reward, and the faster was the reaction time, suggesting that any behavioral differences were only reflected in the inverse gain rather than in the learning rates. However, additional exploratory analyses revealed that the learning rates captured dynamical aspects of choice behavior: while the learning rate for gain correlated with collected reward the highest during early trials (*b*_*0*_ = 0.301, *p* < 0.001; *b* = − 0.004, *p* < 0.001), the correlation between learning rate for loss increased towards later trials (*b*_*0*_ = 0.046, p = 0.020; *b* = 0.001, *p* < 0.001), The traces of correlations throughout the task are depicted in Fig. 2 (bottom).

### Explicit treatment expectations on symptoms improvement correlate with purported treatment efficacy but not RL parameters

We exploratively assessed if our expectation manipulation elicited responses in explicit expectation ratings analogous to the quadratic pattern in learning rates for gain. We used three items of the *Generic rating scale for previous treatment experiences, treatment expectations, and treatment effects*^45^ (GEEE) separately asking for expected learning performance improvement, worsening from the treatment as well as expected side-effects from the treatment (see Methods). An additional item asked for the subjective certainty of performance improvement ranging from *certainly ineffective* to *certainly effective*. Bayesian regression models with the expected treatment efficacy as predictor and each questionnaire item as dependent variable were tested against an intercept-only model and for the model with the largest Bayes factor, explained variance and model significance are reported. The results yielded anecdotal evidence (1 < BF_10_ < 3) for a combination of linear and quadratic effects for expected performance improvement (BF_10_ = 2.69; group-only model: BF_10_ = 1.61; quadratic-only model: BF_10_ = 0.73), which explained *R*^*2*^_*adj*_ = 4.9% of the variance (*p*_*model*_ = 0.012). These results implied maximum (explicit) treatment expectations on symptoms improvement in a range between purportedly maximum certain and uncertainty treatment efficacy. There was further very strong evidence (BF_10_ > 10^3/2^) for a linear effect on expected certainty of positive treatment efficacy (BF_10_ = 74.7, *R*^*2*^_*adj*_ = 8.2%, *p*_*model*_ < 0.001; model including group and quadratic term: BF_10_ = 64; quadratic-only model: BF_10_ = 0.35) suggesting that participants in groups with higher provided treatment efficacies also expected a more certain improvement in learning performance. There was no evidence for effects of purported probability of treatment efficacy on expected symptoms worsening and expected side effects count (all BF_10_ < 0.45), except for anecdotal evidence for a quadratic effect on expected symptoms count (BF_10_ = 1.28,* R*^*2*^_*adj*_ = 0.1%, *p* = 0.273). This translated to a specific variability in positive, rather than negative, treatment expectations caused by the experiment. See Supplementary Fig. S4 for participants’ ratings on GEEE expectation scale, separately for each group and item.

Additional Spearman’s rank correlations between all questionnaires used in this study and RL parameters are shown in Supplementary Fig. S5. With respect to effects of personality traits, we observed a significant correlation between the learning rate for loss and the BFI-10 neuroticism scale (*r* = − 0.19, *p* = 0.028).

### Forgetting might contribute to observed effects of treatment uncertainty

We performed additional analyses, since a large portion of behavioural effects has been reflected in the inverse gain parameter capturing decision noise. To this end, we refined the previously analysed model and tested it against two different candidate models.

**Figure 5:**
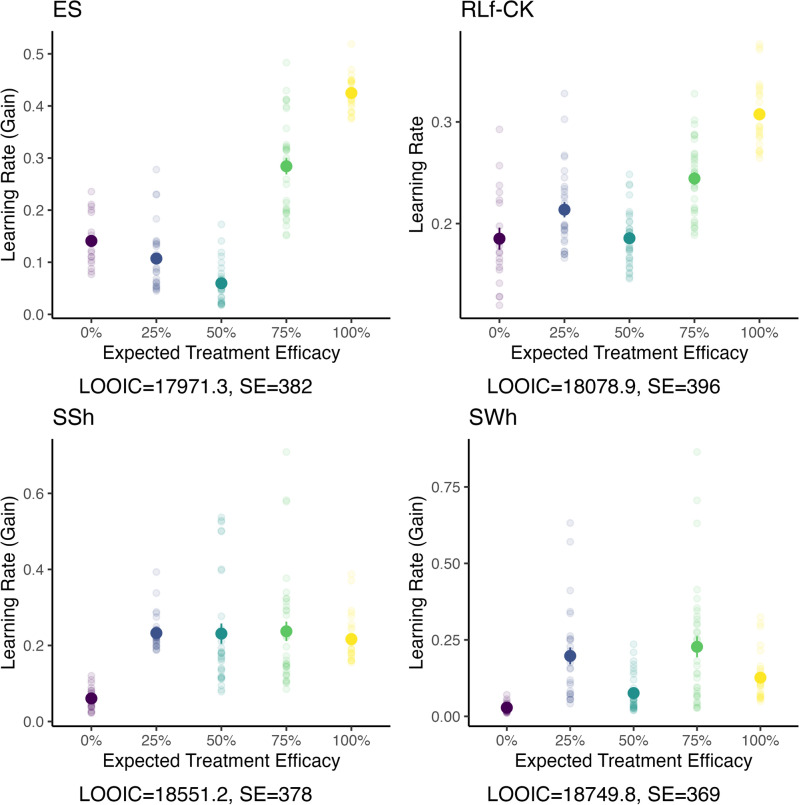
Learning rates per treatment condition across four compared models. Learning rates of each model, either for gain or general updating, are depicted on the y-axis. Purported probabilities of treatment efficacy are denoted on the x-axis. Bold dots represent group-level means, and error bars indicate the standard error. Models are sorted from lowest to highest LOOIC, from top left to bottom rights. LOOIC and its standard errors are shown below the respective figure cells. We compared an extended standard model (ES; top left) with a forgetting-RL model including a choice kernel component (RLf-CK; top right), the translated standard model with small priors using the *hBayesDM* R package (SSh; bottom left) as well as its variant with wide priors (SWh; bottom right).

First, we tested the robustness of the previously analysed standard model with wide priors and the original sampling procedure (*SWo model*) by narrowing the priors (*SSo model*; see Methods), revealing a similar treatment effect pattern as previously reported (see Supplementary Fig. S6 for individual posterior means), but exhibiting less within-group variance. A monotonic pattern was observed in 4.3% and 41.0% of the posterior samples for the strong and weak monotonicity hypothesis, respectively, and 17.2% and 57.8% for the inverted-U hypothesis. Further, we translated the SSo model to the *hBayesDM* framework in R^46^ (*SSh model*; Fig. 5, bottom left), which per default sets initial values using a variational Bayesian approach, using freely available scripts (https://github.com/CCS-Lab/hBayesDM). In both the SSh and the SSo model, the learning rates for gain revealed similar patterns to the SWo model. Widening the priors again, on the contrary, disrupted the inverted-U-pattern (*SWh model*; Fig. 5, bottom right).

Second, we conducted a model comparison between the SSh and SWh model, consisting of two separate update parameters and an inverse temperature parameter (i.e., the inverse of the inverse gain parameter used in the SSo and SWo model); an extended standard model (*ES model*) similar to the SSh model, additionally including a forgetting rate^47^, irreducible noise^48^, and an initial bias^49^; and a forgetting-RL model with only one update and inverse temperature parameter each, and a choice kernel component^50^ (see Methods for all parameter definitions). Hierarchical model comparison was performed using a fully Bayesian LOOIC^51^ applying the function *hBayesDM::extract_ic* to the *hBayesDM* model objects fitted across all participants irrespective of the treatment groups. The lower the LOOIC of a model, the better its out-of-sample prediction accuracy. The ES model yielded the lowest Leave-One-Out Information Criterion score (LOOIC = 17,971, *SE* = 382), closely followed by the RLf-CK model (LOOIC = 18,079, SE = 396) and, to a lesser extent, the SSh model (LOOIC = 18,551, *SE* = 378) and the SWh model (LOOIC = 18,750, *SE* = 369). To test for differences of the sampling procedure, a prediction accuracy score was computed for the SSo without group constraints. The analysis yielded the lowest score for this model compared to all other models (LOOIC = 17,906, *SE* = 394), supporting the quality of our initial model.

Additional parameter recovery was performed for all *hBayesDM* models fitted across all participants by extracting the individual group-level parameter posterior means, simulating new choice data for N = 141 agents over 120 trials each, and correlating the obtained parameters for simulated data with the generative RL parameters. Given the close to identical LOOIC for the ES and RLf-CK model, we considered the RLf-CK model as the better performing model due to a higher average Spearman’s rank-correlation coefficient for simulated vs. obtained parameters (*r*_ES_ = 0.46, and *r*_RLf-CK_ = 0.66), and parsimony (five instead of six parameters). The SSh model yielded an average recovery correlation of *r*_Spearman_ = 0.07, indicating parameters did not recover well using this procedure.

As part of the hierarchical comparison, all *hBayesDM* models were fitted separately for each group and tested for differences in the estimated parameters. The unified (non-separated) learning rate parameter of the RLf-CK model revealed a reverse updating pattern in the first place: The learning rate now exhibited a U-shape, instead of an inverted-U-shape, indicating the slowest updating from recent prediction errors (Supplementary Figure S7). Importantly, the introduced forgetting rate mirrored this pattern, indicating the least forgetting under maximum treatment uncertainty. This could suggest the inverted-U-shape of learning rates for gain in the SWo model to be equally reflected in reduced forgetting and updating. We tested this assumption by regressing the learning rates for gain, and inverse gain, on all parameters of the RLf-CK model. Our findings revealed a multiple *R*^2^ = 18.3% for the learning rates for gain, significantly accounted for by all RLf-CK parameters (all *p*_Bonferroni_ < 0.024), except for forgetting (*p*_Bonferroni_ = 0.473). Instead, forgetting rate and choice inverse temperature predicted inverse gain of the SWo model (both *p* < 0.001), and the regression model explained *R*^2^ = 70.6% of the variance in inverse gain. On one hand, this suggests the SWo decision noise largely to be accounted for by a forgetting process; on the other hand, no significant explanatory power of the forgetting rate regarding the learning rates for gain supports the assumption that both models might provide independent formulations of potential treatment targets.

Last but not least, we tested which parameters of the RLf-CK model predicted collected reward in order to understand the contribution of a potential treatment target. Only the forgetting rate significantly predicted collected reward, as indicated by standardized regression coefficients of a generalized linear mixed model ($\beta$ = − 0.13, 95% CI [− 0.18, − 0.08]), suggesting less forgetting in particular to promote successful collection of reward throughout the task.

## Discussion

In our study, we investigated how varying degrees of expected treatment efficacy affect reward sensitivity and compared two hypotheses assuming either monotonicity or an inverted-U-shape relationship. For this purpose, we contrasted a monotonic and an inverted-U-shaped pattern of reward sensitivity after an auditive sham treatment using binaural beats. We collected data in an online experiment, in which participants listened to binaural beats for three minutes and then performed a probabilistic reinforcement learning task. Crucially, participants were, unknown to them, separated into five groups differing with regard to the provided probability of treatment efficacy ranging from 0 to 100 percent in steps of 25 percent.

Our main findings indicated an inverted-U-shaped pattern of the learning rates for gain dependent on the expected treatment efficacy. In context of prediction-error driven updating (e.g., Rescorla-Wagner updates or RL), the learning rate determines the degree to which the outcome of an event, such as a choice in a probabilistic reinforcement learning task, influences the built expectation towards the outcome of a succeeding event. The higher a learning rate is, the more the current expectation relies on more recent outcomes. The highest learning rates for gain under maximum reward uncertainty observed here thus imply the strongest weighting of the most recent reward in the maximum uncertainty group with a provided 50 percent probability of treatment efficacy. While a higher learning rate for gain enables the agent to adjust reward expectations in a variable environment more appropriately, it might also lead to an overfitting to random fluctuations in a stable environment, which in the long run reduces collected net reward: for example, if a certain choice is rewarded 70 (vs. 30) percent of the time, consistent choice of this option results in 70 percent reward, whereas occasional switching after incorrect feedback would result in a total reward below 70 percent. In this regard, we could show that correlations with behavioral performance appeared to depend on the trials considered. While the learning rate for gain exhibited higher correlations with collected reward during early trials, the learning rate for loss showed the highest correlations with collected reward during late trials (Fig. 2e). Therefore, higher learning rates reflected faster behavioral optimization towards reward during early trials of the task, and particularly under maximum uncertainty of treatment efficacy.

In contrast, additional analyses involving a choice kernel component and forgetting rate parameter suggested a forgetting process as a complementary explanation for the data, exhibiting a U-shaped curve and accompanied by a U-shaped treatment effect for general RPE updating, with least forgetting and updating observed under maximum treatment uncertainty. In this regard, reduced forgetting, but not general RPE updating was significantly associated with more overall collected reward. In line with the notion of Frank et al.^43^, a low learning rate during training would benefit accurate learning of reward probabilities, as opposed to rapid adaptations. Therefore, both the SWo and the RLf-CK model might hint at more pronounced feedback integration under maximum treatment uncertainty with i) *higher stability of feedback integration* as indicated by reduced forgetting and sensitivity to feedback (learning rate), but ii) an *increased sensitivity to rewards*, while iii) learning rates for loss were small in general, enabling a *more stable integration of losses*. However, due to the observed differences in the predictive model accuracy, the results of alternative models should be taken with caution.

RL parameters may indeed show sensitivity to dopaminergic treatments^52–54^. However, interpretability and generalizability of RL parameters still require caution as effects may vary dependent on performed task and computational model used^55^. Considerable interindividual variability can be seen in Fig. 3, which may reflect true individual differences in learning rates, or could hint at an incorrect model. After recovering the choices as a function of the simulated choice probability conditioned on observed and simulated data, the model’s choices constituted a mostly accurate and weakly biased representation of the participant’s data (Fig. 4): the visual correlation of the model’s and participants’ percentage of optimal choices as well as likelihood curves contrasted to observed optimal choices suggest a reasonable match between both the model and participants’ decisions, and parameters did not exhibit visual correlations within groups. While our choice for the considered model was based on extensive validation in the literature^43^ and did not control for additional effects, such as choice-autocorrelation posing the risk of estimation biases^56^, it exhibited the best predictive accuracy in this study. The introduced stay probability as a conventional and model-neutral alternative supported separate learning rates for gain and loss. Nevertheless, aims to replicate these findings as well as future extensive model comparisons are highly desired in order to reveal the actual parameters targeted by the treatment.

Under the assumption that our estimates of the extensively analyzed (SWo) model reflected genuine reward sensitivity, the results support a potential link between the placebo effect and the pattern of dopamine release and firing usually observed under different (conditioned) reward probabilities, although the true cognitive and neural processes involved could not be revealed in this study. In search of mechanistical explanations for the observed effects of uncertainty in context of placebo treatments, and to motivate further research in this regard, we would speculate that induced treatment uncertainty could have broadened the subjects’ probability distribution of possible treatment outcomes, which would bind attentional resources in order to understand how sensory input, i.e., the purported reward learning enhancement, is generated^57^. We would therefore speculate that (1) while other sensations indicating treatment efficacy (e.g., bodily symptoms) might have been uncertain, the potential search for efficacy-confirming information (here: the task reward as a subjective indicator of *reward learning enhancement* purported by the instructions) could have helped at improving updating from positive task feedback^58^, and that (2) increased attention as only one of other potential cognitive process involved under uncertainty^26^ might have additionally influenced RL parameters^59^. More broadly, the instruction of maximum treatment uncertainty might have engaged participants in finding out if the treatment actually works or not, with obtained task rewards as a potential subjective indicator of efficacy. If confirmative or disconfirmative information is sought under uncertainty, tuning reward detection towards a more liberal signal detection criterion would be beneficial for reward seeking^60^, with positive effects also observed in placebo analgesia^61^. Therefore, the observed elevated strength of positive feedback processing indicates a potentially beneficial effect of uncertainty in a placebo treatment.

As a limitation of our RL results, while frequentist ANOVAs yielded significance for the effects of expected treatment efficacy on individual RL parameter posterior means, there was no clear evidence of either an effect or indifference regarding individual posterior means of learning rates for gain and the inverse gain parameter, as obtained by the Bayesian ANOVAs. The frequentist results may therefore be an artifact of overfitting. In contrast, a Bayes factor of 80.8 for learning rates for loss constitutes very strong evidence for an actual effect. Post-hoc comparisons revealed that for the learning rates for gain, as the RL parameter of interest, the contrast between 0 and 50% expected treatment efficacy drove the group effect, supporting the role of treatment uncertainty in increasing reward sensitivity. As a second consequence, our findings could suggest the learning rate for loss as a more certainly affected index of RL in context of efficacy manipulation compared to the learning rate for gain. On the transformed (inverse logit) scale, however, learning rates for loss close to the lower limit of the sampling boundaries may indicate a floor effect (see Fig. 2 and Supplementary Fig. S1) posing the risk of driving correlations though influential data points and making interpretation difficult. This points at the need for further studies addressing effects of variations in expected treatment efficacies involving loss-oriented RL conditions, or more sophisticated RL models, so that more reliable estimates for loss sensitivity could be taken into account. Further, flooring learning rates for loss could implicate that our task may not have actively engaged participants in avoiding no rewards, but rather in collecting rewards (i.e., *positivity bias*), which would support the importance of the learning rate for gain as the primary parameter involved in the performed probabilistic RL task.

Moreover, we included measurements of participants’ explicit expectations by means of the GEEE questionnaire items in our analyses to further understand in which manner expectations would affect RL behavior. If the uncertainty-driven reward sensitivity increase was based on explicit expectations, learning rates for gain and the provided probability of treatment efficacy should have had correlated. However, although our experimental manipulation evoked differential expectation ratings, we found no significant correlations between any GEEE item and any RL parameter, providing further support for the postulate that explicit expectation ratings are limited in predicting the placebo response^62^. As an important limitation of these results, we did not measure participants’ beliefs regarding the instructions at the end of the study, which would have allowed us to regress these beliefs out of the results, and we also did not correct for multiple testing of correlations due to the exploratory nature of the analysis, which requires caution at interpreting. For the experimental manipulation of inducing treatment uncertainty, we used numerical probabilities of efficacy supported by a verbal expression of probability with the goal to make the instructions more understandable, which at the same time might render the observed effects of provided probability of treatment efficacy here not perfectly linearly interpretable due to subjective variations and shifted means of the targeted probabilities^63^.

Recent research has identified the contribution of expectations to therapeutic enhancement, particularly in the domain of pain and depression. While placebo analgesia is well elaborated, less is known of placebo effects in reward sensitivity, which are theoretically highly relevant for an understanding of depressive disorders^19^ (but see *Nielson *et al*.*^64^ for a critical review). Both areas show a common reliance on dopamine as a central neurotransmitter of reward signaling. In clinical practice, maximum certainty of efficacy is considered to evoke the largest therapeutic effect. However, if placebo-induced dopamine enhancement is involved in reward sensitivity according to the placebo-reward hypothesis, reward uncertainty as a promoter for dopamine release and firing may ultimately come along with a greater placebo response in this regard. A previous study^37^ had addressed this issue and, indeed, found increased reward sensitivity after high vs. low uncertainty of provided treatment efficacy.

Our study may shed new light on the role of treatment uncertainty in placebo interventions. We showed that eliciting maximal uncertainty regarding the treatment efficacy enhanced an index of reward sensitivity, which followed an inverted-U pattern as a function of the provided probability of treatment efficacy. The pattern exhibited here bore striking resemblance with the neural signature involving dopamine under uncertainty, but the exact mechanisms involved in the observed performance increase remain open for debate. If our findings as well hold for a clinical sample, a placebo treatment aiming at increased reward sensitivity may thus help improving the treatment of major depressive disorder^19,65–68^ by systematically adding uncertainty to the expected treatment efficacy. A stronger weighting of recent (positive) experiences as reflected in a higher learning rate for gain under maximum treatment uncertainty might therefore add to therapeutic benefits. Future studies could try to address this topic under discomforting symptoms in clinical populations: while our current investigation on placebo effects under uncertainty relied on reward-associated features, we assume that uncertainty also comes into play at learning from negative task or symptom outcomes. For example, trait anxiety is thought to be particularly sensitive to uncertainty and could be linked to more pronounced learning from negative outcomes under stress^69^. Therefore, it would be presumable that under anxiety and stress, the inverted-U-shape in reward sensitivity would attenuate in terms of a shift towards learning from loss. Of interest might also be the temporal dynamics of reward sensitivity increasing placebo interventions and whether there are time periods particularly sensitive to this effect: is reward sensitivity increase sustained over the course of the treatment, or are they specific to a certain period of high uncertainty about the treatment efficacy? Also, would repeated exposure to a placebo treatment still support increased reward sensitivity in order to induce long-term symptoms improvement?

Our findings serve as first supporting evidence of the contribution of uncertainty to increased reward sensitivity under positive treatment expectations. Further, the approach used here increased the resolution of observed treatment effects by operationalizing uncertainty by means of standardized definitions of verbal efficacy probabilities. The online-based implementation will allow placebo research outside the lab enabling the collection of large samples. Inducing uncertainty in placebo interventions could help boosting updating from rewards, thus presumably facilitating the incorporation of positive event outcomes into expectations and future behavior, thereby ameliorating potentially reduced reward sensitivity observed in depressive disorders. Translational studies harnessing these findings into practice and aiming at understanding what neural underpinnings exactly are responsible for the uncertainty-related placebo effect on reward sensitivity, are therefore highly desirable.

## Methods

### Participants

We recruited 143 participants (57 male, 1 diverse) with a mean age of 29.3 years (*SD* = 13.3 years) using the local research participation system of the University of Marburg and by direct personal approach. All participants were reportedly German native speakers with a minimum age of 18 years and current residency in Germany. A self-reported history of neurological disorders, particularly epilepsy or stroke, and mental disorders within the last three years precluded from participation. Two participants, who responded less than three times the standard deviations subtracted from the samples mean response count, were excluded resulting in a final sample size of 141. For correlations involving questionnaire data, one participant was additionally excluded due to failing to answer a control item correctly. Participants gave their informed consent and were reimbursed with course credits and feedback on provided questionnaire data. The study was conducted between May and June 2021 and was approved by the Local Ethics Committee of the University of Marburg Psychology Department (2021-38k). The study was conducted in accordance with the Declaration of Helsinki.

### Paradigm

Participants performed the training phase of a probabilistic reinforcement learning task^70^ programmed in *jsPsych*^71^ (version 6.2.0). The task comprised six black Japanese hiragana letters grouped into three fixed pairs with different reward probabilities on a white screen (Fig. 1, bottom). Participants had 1700 ms per trial to press ‘N’ or ‘M’ in order to make a choice between both stimuli of a pair and were subsequently presented a probabilistic feedback screen showing a green circle (gain) or a red cross (loss) for 1000 ms (Fig. 1, top). The task included 40 trials per letter pair resulting in a total of 120 trials per participant in randomized order. Maximal task duration was, therefore, eight minutes. The trials were balanced regarding the stimulus position on the screen. Eighteen training trials of the probabilistic RL task with Kanji character stimuli were presented to make participants familiar with the task.

### Experimental manipulation

All participants were randomly assigned into five groups through a random generator in the experiment’s code, which allows unbalanced group sizes. The groups varied with regard to the provided therapeutic efficacy instruction as probability from 0 to 100 percent in steps of 25 percent (“In recent studies, an improving effect on learning performance could be observed in (a) none of all, (b) 1 out of 4, (c) half of the, (d) 3 out of 4, (e) all participants. Therefore, an improvement of learning is (a) impossible, (b) unlikely, (c) uncertain, (d) likely, (e) certain.”).

### Binaural beats

The presented binaural beats were played for 183 s to all participants, regardless of condition, and served as sham treatment. We used tones of 160 Hz and 180 Hz separately on each ear with a ten seconds fade-in and fade-out. We decided for binaural beats as treatment, as there is no clear evidence for any effects after short-term exposure^42,72,73^ while presumably inducing treatment-related sensations.

### Questionnaires

All questionnaire items were displayed in German. Participants were asked to provide their age, sex, handedness, cigarettes consumption, whether they studied psychology at that time and if they had even marginal knowledge of Japanese symbols. The expectation scale of the *Generic Rating Scale for Previous Treatment Experiences, Treatment Expectations, and Treatment Effects*^45^ (GEEE) was used in order to assess treatment expectations. This scale asks for participants’ expected improvement, worsening and count of side effects from a particular treatment on a particular outcome (here: “How much (1) improvement/(2) worsening of learning performance do you expect from the treatment with binaural beats?”; “How many complaints/side effects do you expect from the treatment with binaural beats?”), which is indicated on a 11-point Likert scale from zero (no improvement/worsening/complaints) to ten (greatest improvement/worsening/complaints imaginable). A self-developed pilot item asking for expected treatment certainty towards positive outcomes (“How sure are you the treatment will have an impact on your learning performance?”) from zero (“certainly ineffective”) to ten (“certainly effective”) with an additional label at five ("uncertain”) was added. We further included questionnaires to potentially assess trait and state personality variables: the *Positive and Negative Affect Schedule*^74^ (PANAS) with four additional items (“expectant”, “sad”, “happy”, “motivated”), which were not considered in this study; the *Temporal Experience of Pleasures Scale*^75^ (TEPS; German translation^76^) for anticipatory and consummatory anhedonia, and the *Big Five Inventory*^77^ (BFI-10), which captures the Big Five personality traits. In addition, a control item („This is a control item to ensure data quality. Please check option ‘6’.”) was added to the TEPS.

### Procedure

First, all participants gave their informed consent. They indicated whether they fulfilled the criteria for participation before answering the BFI-10 first, followed by TEPS and PANAS. Afterwards, the participants read one of the five deceptive instructions on the treatment efficacy of binaural beats (see Experimental Manipulation). In order to make sure they had read the whole instruction set, participants were required to press a specific button on their keyboard that was mentioned in the last sentence on the screen. Participants were required to use their headphones. To make the auditive treatment more comparable across the whole sample, a set of instructions assisted the participants in putting the headphones into the respective ears and to adjust the volume to a pleasant level, before listening to the binaural beats. Participants, who tried to skip the audio manipulation by manipulating the source code, were automatically excluded at this stage through a preventive mechanism in the code. After the audio manipulation, a set of instructions for the probabilistic RL task was presented. Participants were informed that they would play a reward task for approx. 10 min and that the task requires learning by trial-and-error in order to identify the more beneficial letter and therefore to maximize reward. Neither monetary nor another mode of reward was mentioned. The participants were made aware of different reward probabilities between the pairs and further of the possibility that also the overall less beneficial choice could occasionally be rewarded. After performing the probabilistic RL task, participants checked a box to disclose as to whether accurate data was provided. Questionnaire scores were provided as feedback at the end.

## Analyses

Data were analyzed in R^78^ (version 4.3.1) using RStan^79^ (version 2.21.8) for computational modeling. Stan models were fitted on an AMD Threadripper 2990WX and analyzed using parts of analysis scripts from *Turi *et al*.*^37^ (https://github.com/ihrke/2016-placebo-tdcs-study), which were adapted for the purpose of our study. A Q-learning model incorporating the Rescorla-Wagner update rule separately for gain and loss was used for estimation of learning rates for gain (*α*_G_) and loss (*α*_L_):

$${Q}_{i}\left(t+1\right)={Q}_{i}\left(t\right)+{\alpha }_{G}{\left[r\left(t\right)-{Q}_{i}\left(t\right)\right]}_{+}+{\alpha }_{L}{\left[r\left(t\right)-{Q}_{i}\left(t\right)\right]}_{-}$$

$${Q}_{i}\in \left[\text{0,1}\right],i\in \{\text{1,2},\dots ,6\},r\in \{\text{0,1}\},\alpha \in \left[\text{0,1}\right]$$

The inner core of Q-learning is a slow integration of trial gains and losses (*r*; 1 for gains, and 0 for losses) into a stimulus-linked expectancy value *Q* of stimulus* i*, which is passed to a decision probability likelihood function for model fitting,

$${P}_{A}\left(t\right)=\frac{{e}^{\frac{{Q}_{A}\left(t\right)}{\beta }}}{{e}^{\frac{{Q}_{A}\left(t\right)}{\beta }}+{e}^{\frac{{Q}_{B}\left(t\right)}{\beta }}},{P}_{A}\left(t\right)\in \left[\text{0,1}\right],\beta \ge 0,$$

where *P*_*A*_ denotes the choice probability for stimulus A over B in a given trial *t*, and *β* the inverse gain parameter that scales the Q-values prior to the softmax operation, was evaluated at observed individual choices. We applied MCMC sampling comprising of eight chains with 10,000 iterations for warm-up and an equal number for sampling with five separate group-level distributions:

$$logit\left({\alpha }_{j}\right)\sim Normal\left({\mu }_{{\alpha }_{0}}+{\delta }_{{\alpha }_{j}},{\sigma }_{\alpha }\right)$$

$$log\left({\beta }_{j}\right)\sim Normal\left({\mu }_{{\beta }_{0}}+{\delta }_{{\beta }_{j}},{\sigma }_{\beta }\right)$$

$$j\in \text{0,25,50,75,100},\; j=0\Rightarrow {\delta }_{{\theta }_{j}}=0$$

$${\mu }_{\theta }\sim Normal\left(\text{0,100}\right)$$

$${\sigma }_{\theta }\sim Uniform\left(\text{0,100}\right)$$

$${\delta }_{\theta }\sim Normal\left(\text{0,3}\right)$$

Here, *μ*_*θ*_ is the group-level mean of each RL parameter *θ*, *σ* denotes the variance, and *δ* means the difference to the baseline group, i.e., 0% treatment efficacy (*j* = 0). The target average acceptance probability and the maximum tree depth were set to 0.99 and 15, respectively.

To assess the model performance, single-trial choices were simulated by re-running the Q-learning algorithm on the participants’ observed choice data as well as simulated task data using the sampled individual posterior means of RL parameters *θ* and compared with observed data (Fig. 4a, b). Here, we defined *model accuracy* as the mean model likelihood for observed choices. Choice simulation on simulated task data was repeated 20-fold due to the involvement of random uniform sampling, i.e., (1) choice of stimulus A, if the computed decision probability for stimulus A exceeded random thresholds in each trial, and (2) random choice of either Stimulus A or B, if both stimuli exhibit equal choice probabilities. The R packages BayesFactor^80^ (version 0.9.12.4.4) and bayestestR^81^ (version 0.13.1) were used for Bayesian testing of group effects on RL parameter means for individual posterior and group-level distributions, respectively.

Group effects on individual behavioral measurements (reaction time, count of optimal choices, collected reward, and stay probability) and model fit, and effects of reward interaction on stay probability were estimated using hierarchical generalized linear mixed-models with participant ID as random effect using the lmerTest package^82^ (version 3.1.3). For reaction times, a gaussian distribution with log-link function was assumed, while optimal choices, collected reward and stay choices as discrete variables on single-trial level were modeled using a binomial distribution. Additional Bayesian estimates for hierarchical testing of these effects were computed using BIC-based Bayes factors^83^ with

$${\text{exp}}\left(\frac{{\text{BI}}{\text{C}}_{{\text{H}}_{1}}\text{-BI}{\text{C}}_{{\text{H}}_{0}}}{2}\right),$$

where H1 posits existence of a group effect compared against an intercept-only model. Correlations of reaction time, relative count of optimal choices, collected reward and stay probability with RL parameters were computed using Pearson’s product-moment correlation.

We analyzed univariate effects of group allocation on normally distributed individual RL parameter posterior means using ANOVAs with Bonferroni-correction for 10 post-hoc t-tests per parameter. Additional Bayesian ANOVAs with Jeffries’ prior provided corresponding Bayes factors. Bayesian regression models with expected treatment efficacy as centered numerical predictor were performed for all GEEE items as dependent variables, and tested for the presence of a linear and quadratic term. If the Bayes factor of one or more possible models vs. an intercept-only model per GEEE item exceeded 1 thus supporting a model including a slope, adjusted explained variance and p-value was reported for the model with the highest Bayes factor. We additionally reported all correlations between questionnaire scores and RL parameters using Spearman’s rank-order correlation for exploratory purposes. Tables were produced using the R packages apaTables^84^ (version 2.0.8) and rempsyc^85^ (version 0.1.5).

For additional analyses, we narrowed the priors of the SWo model from $Normal\left(\text{0,100}\right)$ to $Normal\left(\text{0,1}\right)$ for ${\mu }_{\theta }$, and from $Uniform\left(\text{0,100}\right)$ to $Normal\left(\text{0,0.2}\right)$ for ${\sigma }_{\theta }$ (SSo model), as set per default in the *hBayesDM* package^46^ (version 1.21). To test the SWo model specifications in the *hBayesDM* framework, priors were then widened to ${\mu }_{\theta } \sim \text{ Normal}\left(\text{0,100}\right)$ and ${\upsigma }_{\uptheta }\sim \text{Normal}\left(\text{0,100}\right)$ back again (SWh model). The ES model consisted of a forgetting rate parameter $\phi$ as part of a decay component,

$$Q\left(t+1\right)\leftarrow Q\left(t\right)+\upphi \left[0-Q\left(t\right)\right], \phi \in \left[\text{0,1}\right]$$

decaying all six Q-values trial-by-trial to initial Q-values of 0. We also introduced an initial bias ${Q}_{0}$ increasing the Q-value of the chosen option within a pair at the first trial:

$${t}_{pair}=1:Q{\left(t=0\right)}_{chosen}\ge Q{\left(t=0\right)}_{unchosen},Q\left(t=0\right)\in \left[\text{0,1}\right],$$

The response function was extended by an irreducible noise parameter $\xi$, which allowed decision noise independently from the difference of Q-values:

$${P}_{A}\left(t\right)=\left(1-\xi \right)*\left(\frac{{e}^{{Q}_{A}\left(t\right)*\beta }}{{e}^{{Q}_{A}\left(t\right)*\beta }+{e}^{{Q}_{B}\left(t\right)*\beta }}\right)+\frac{\upxi }{2}$$

$${P}_{A}\left(t\right)\in \left[\text{0,1}\right],0\le \beta \le 20$$

The random choice probability in case of two competing stimuli is P = 0.5, thus, $\xi$ multiplied by 0.5 is the weighted probability of *irreducible* decision noise in each trial. Note, that in this model as well as in the following model, $\beta$ denotes the inverse temperature parameter, which is multiplied by the respective Q-values, whereas $\beta$ as inverse gain parameter of the SSo and SWo model divides the Q-values. The RLf-CK model includes a forgetting rate parameter and a choice kernel component controlling for autocorrelated choices:

$$CK{\left(t+1\right)}_{chosen}=CK\left(t\right)+{{\upalpha }}_{CK}*\left[1-CK\left(t\right)\right]$$

$$CK{\left(t+1\right)}_{unchosen}=CK\left(t\right)+{{\upalpha }}_{CK}*\left[0-CK\left(t\right)\right]$$

$${\alpha }_{CK}\in \left[\text{0,1}\right], CK\in [\text{0,1}]$$

Here, choice kernel values CK are updated by a choice learning rate ${{\upalpha }}_{\text{CK}}$ in each trial. Whenever a stimulus is chosen, its CK-value is positively updated, and the CK-value of the competing stimulus is negatively updated. Similar to the inverse temperature parameter, a choice inverse temperature parameter $\tau$ is multiplied by the choice kernel values in the response function to soften the choice kernel-driven gradient in the probability function:

$${P}_{A}\left(t\right)=\frac{{e}^{{Q}_{A}\left(t\right)*\beta +CK_{A}(t)*\tau }}{{e}^{{Q}_{A}\left(t\right)*\beta +CK_{A}(t)*\tau }+{e}^{{Q}_{B}\left(t\right)*\beta +CK_{B}(t)*\tau }}$$

$${P}_{A}\left(t\right)\in \left[\text{0,1}\right],0\le \beta \le 20, -5\le \tau \le 5$$

To investigate the effect of RLf-CK model parameters on reward collection, we computed a generalized linear mixed model with single-trial reward as the criterion, individual RL parameter estimates as the independent variables, and a random intercept per subject ID. Please note, that this model includes neither an irreducible noise parameter, nor an initial bias parameter.

All models considered for LOOIC-based model comparison as well as the SSo model were sampled with 8000 iterations per chain (4000 warm-ups, 8 chains) for the estimation of posterior means.

### Supplementary Information

Supplementary Information.

## Data Availability

Data and code for running the online survey and task, and the analyses are published under Creative Commons Attribution 4.0 license on the University of Marburg Research Data Repository, *data_UMR* (10.17192/fdr/193).
