# Supplementary material for: Uncertainty of treatment efficacy moderates placebo effects on reinforcement learning
Source: Sci Rep. 2024 Jun 22;14:14421. doi: 10.1038/s41598-024-64240-z (PMC11193823; doi:10.1038/s41598-024-64240-z)
Supplement: Supplementary file 1 — Supplementary Information. [file 41598_2024_64240_MOESM1_ESM.pdf]

Supplementary Material

Title

Uncertainty of treatment efficacy moderates placebo effects on reinforcement learning

Authors

Nick Augustat*,

Dominik Endres, &

Erik M. Mueller

Department of Psychology, Philipps University of Marburg, Marburg, Germany

*Corresponding author: nick.augustat@staff.uni-marburg.de

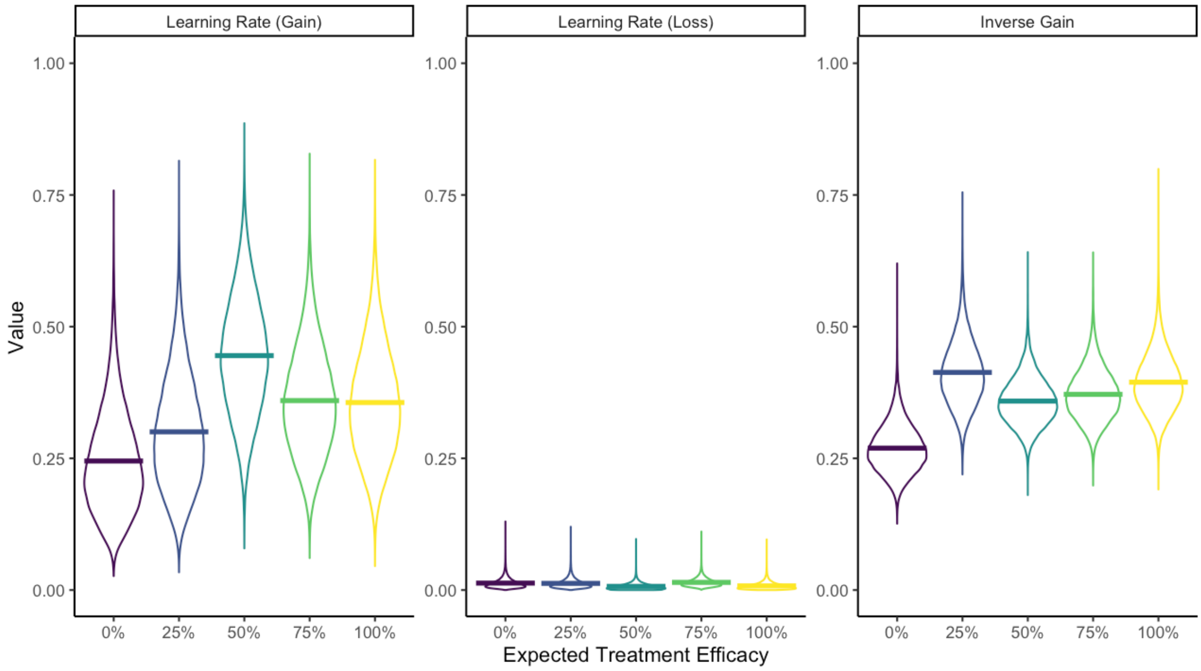

Supplementary Figure S1. Posterior group-level distributions of RL parameters. For each expected treatment efficacy group (x-axis), separate group-level means were estimated via MCMC (y-axis). Crossbars indicate posterior group-level means. The values for learning rates (left, middle) were transformed to logit-normal distributions via the inverse-logit. Inverse gain parameter values (right) were transformed to log-normal distributions using the exponential function.

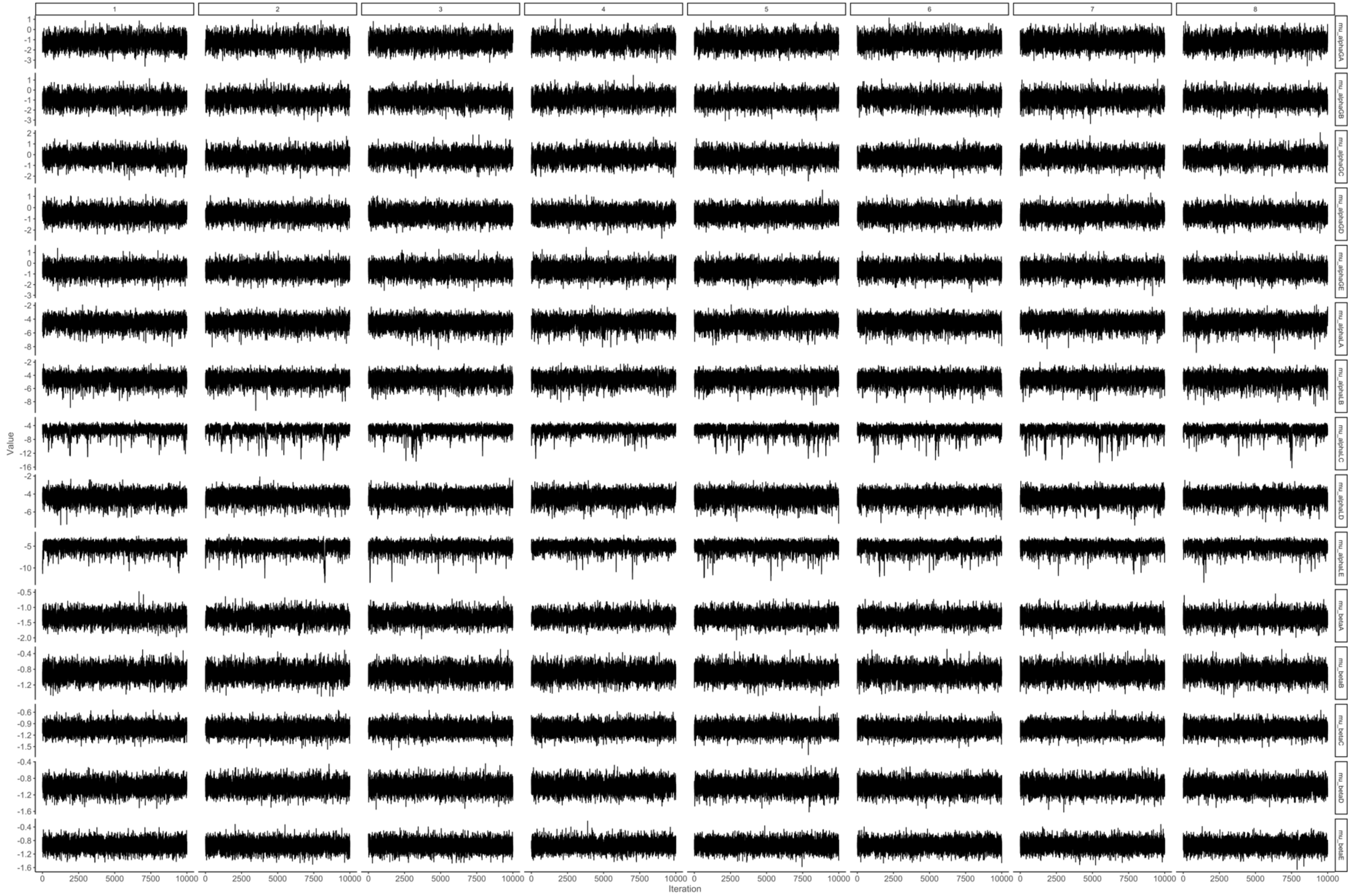

Supplementary Figure S2. MCMC traces for all group-level RL parameters, separately for each expected treatment efficacy group.

Sampled MCMC estimates (y-axis) are shown for iterations from 1 to 10000 for each chain (x-axis).

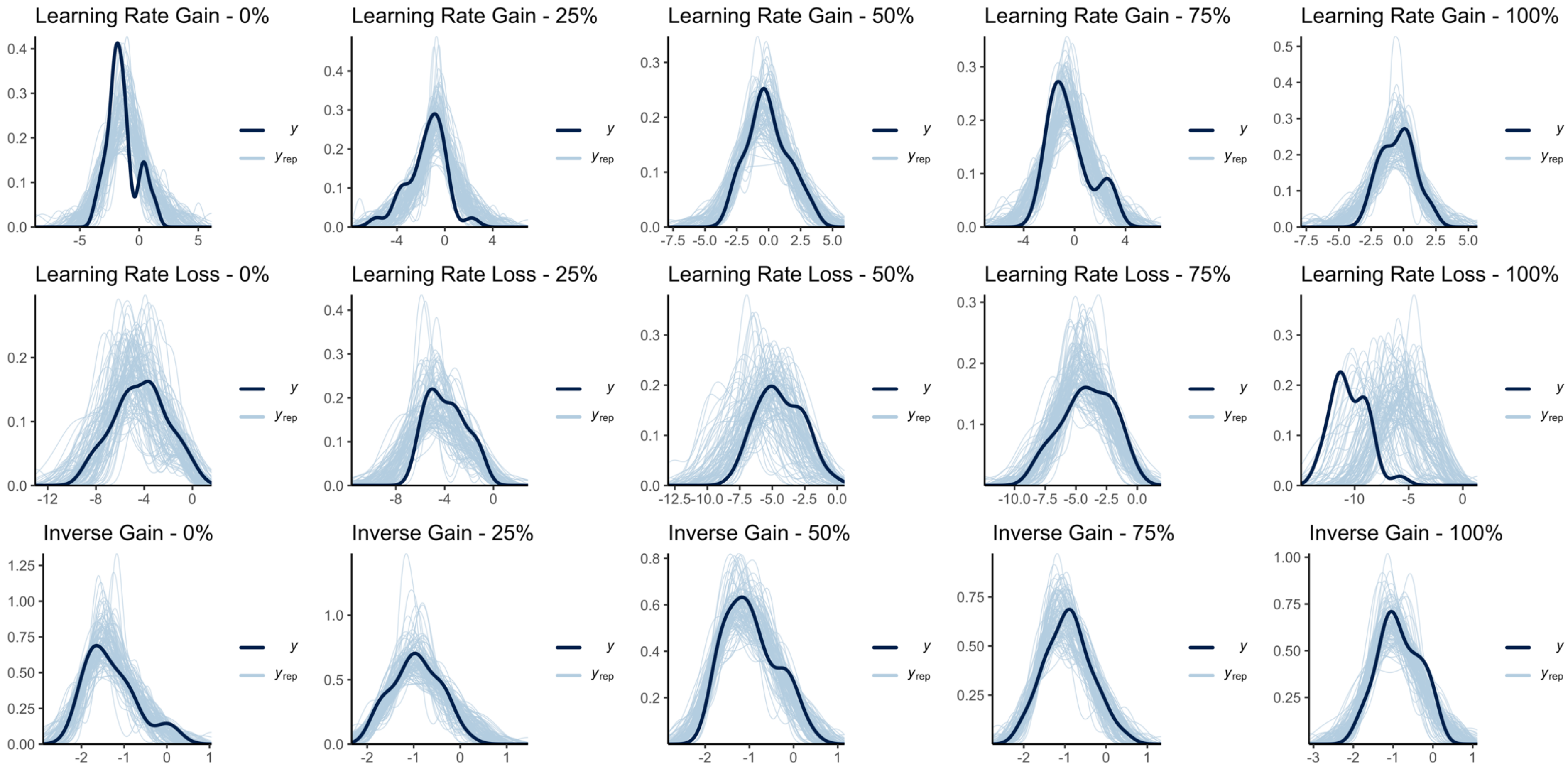

Supplementary Figure S3. Posterior predictive distributions for all RL parameters and groups. Dark blue curves represent the posterior density of the original data, and light blue curves indicate posterior density curves for N=100 predicted replicates using the obtained RL parameter estimates. Parameter values (in logit-/log-normal space) are depicted on the x-axis. The larger the overlap between the light and dark blue curves, the more likely a model constitutes the generative model of the observed data.

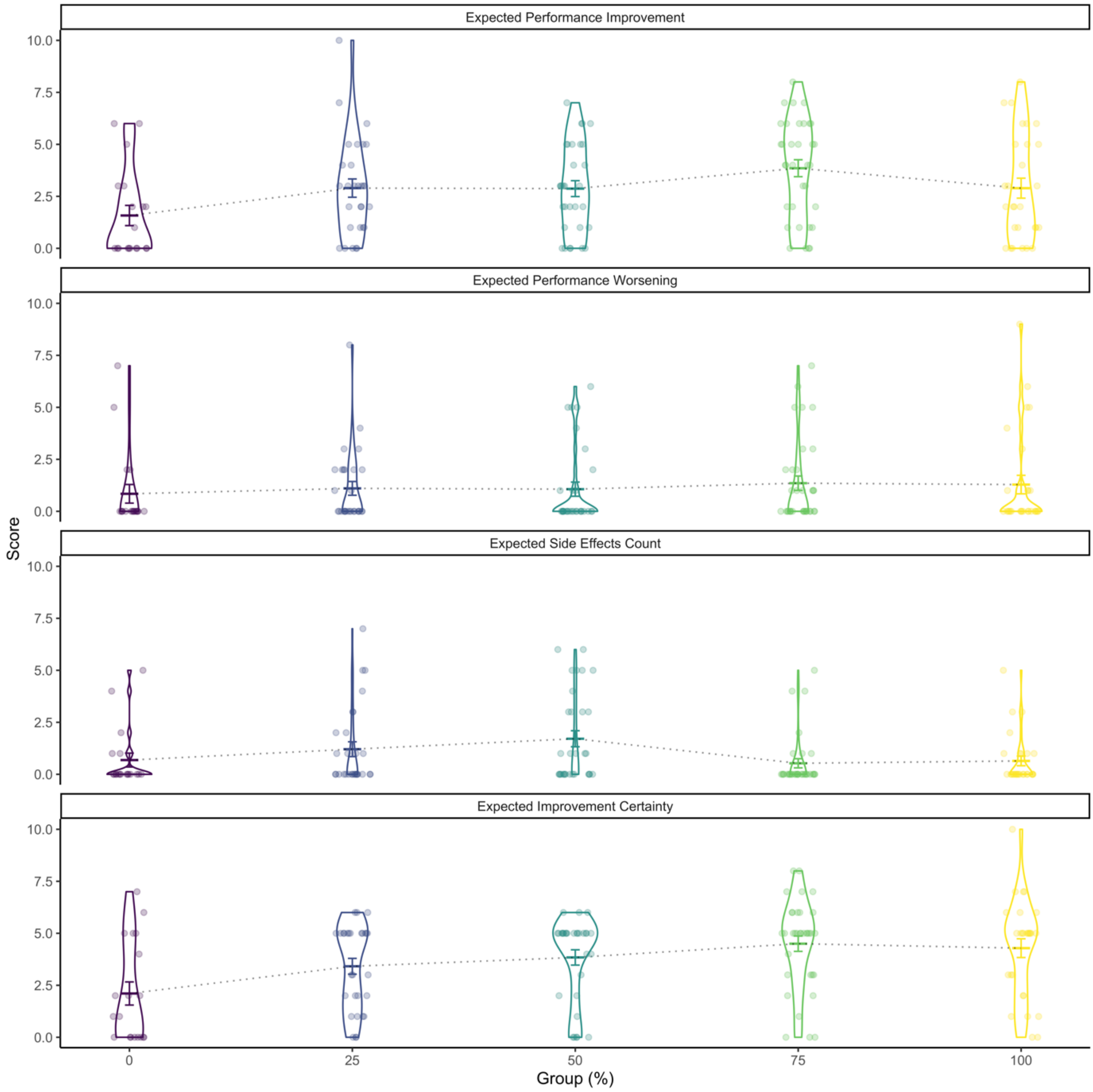

Supplementary Figure S4. GEEE ratings separately for each expected treatment efficacy group. Horizontally jittered participants' ratings and group-level densities (y-axis) are depicted for each group (x-axis). Error bars indicate standard errors, and thick horizontal bars show the group-level means connected through a dotted line.

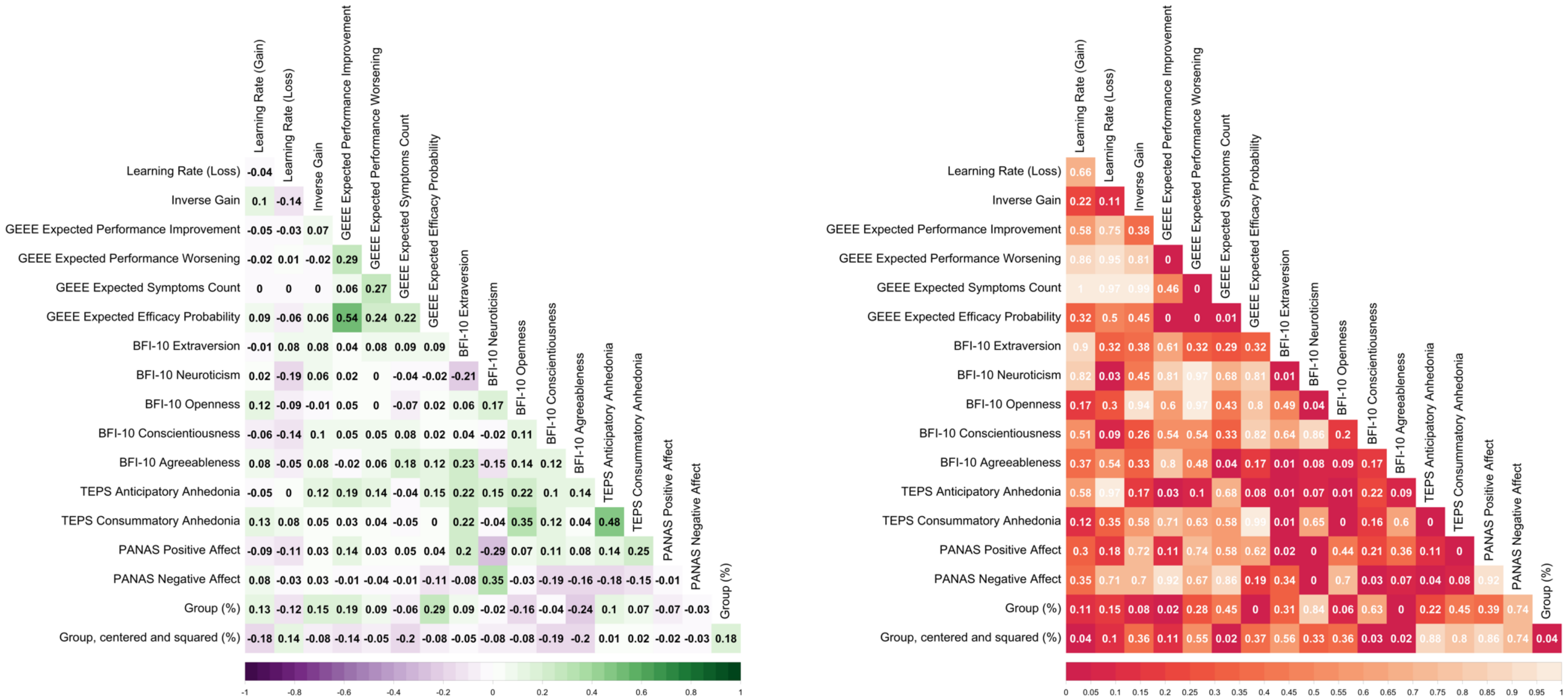

Supplementary Figure S5. Full bivariate correlations across RL parameters and questionnaires. Spearman's rank correlation coefficients (left) and respective p-values (right). Note. Group is the numerical value of instructed treatment efficacy (0-100% in steps of 25%).

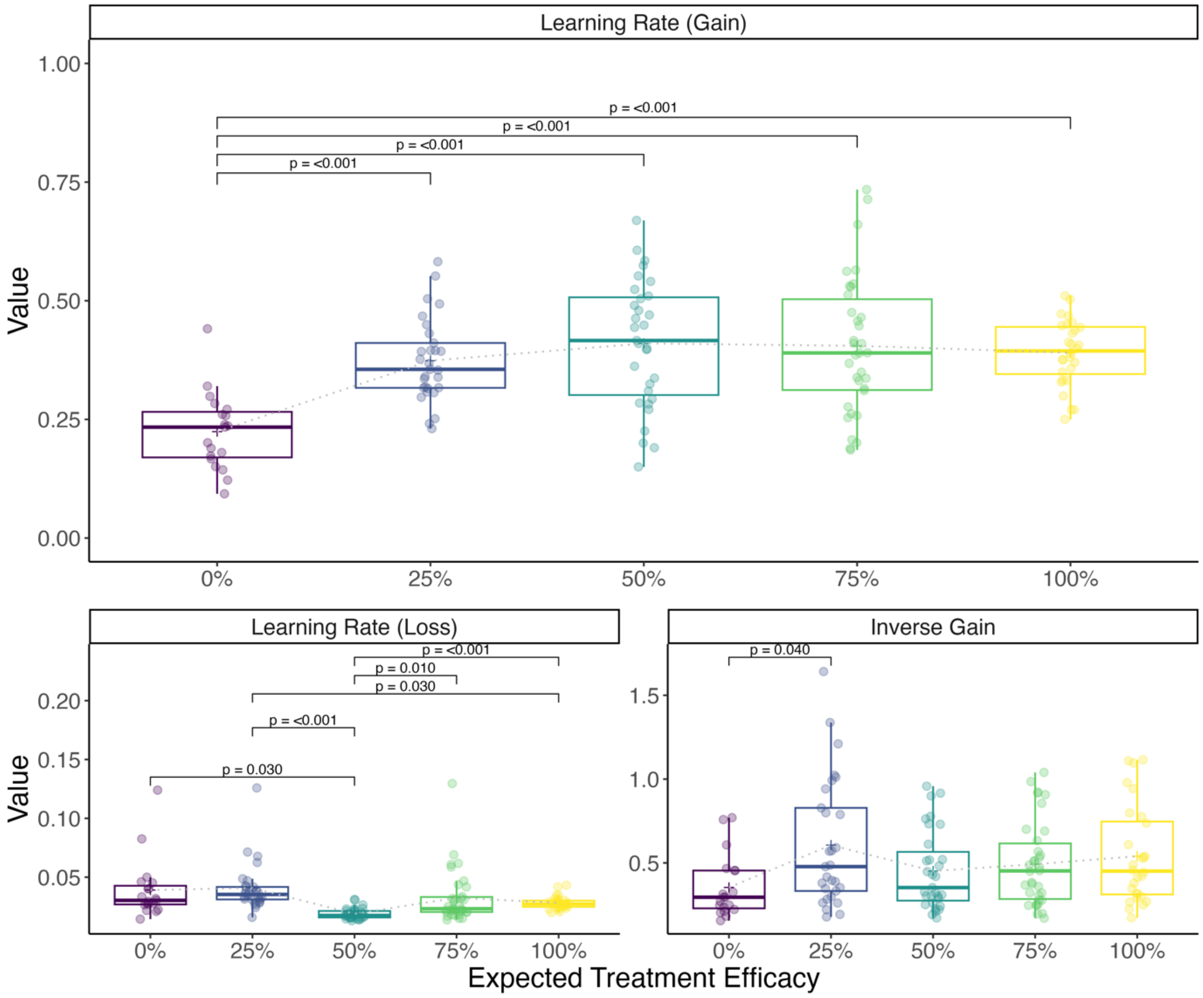

Supplementary Figure S6. Individual RL parameters of the SSo model, separately for each treatment group. The inverted-U-shape for learning rates for gain remains stable after adjusting priors to $\mu_\theta, \sigma_\theta \sim Normal(0{,}100)$. Treatment groups are represented on the x-axis, and transformed (constrained) individual parameter posterior means are shown on the y-axis. Boxplots represent the group-level median (thick horizontal bar), quartiles and whiskers (1.5*IQR). Group-level means are depicted as a cross and connected with a thin dotted line. P-values are displayed, if Bonferroni-correction for 10-fold comparisons was significant at $p < .05$.

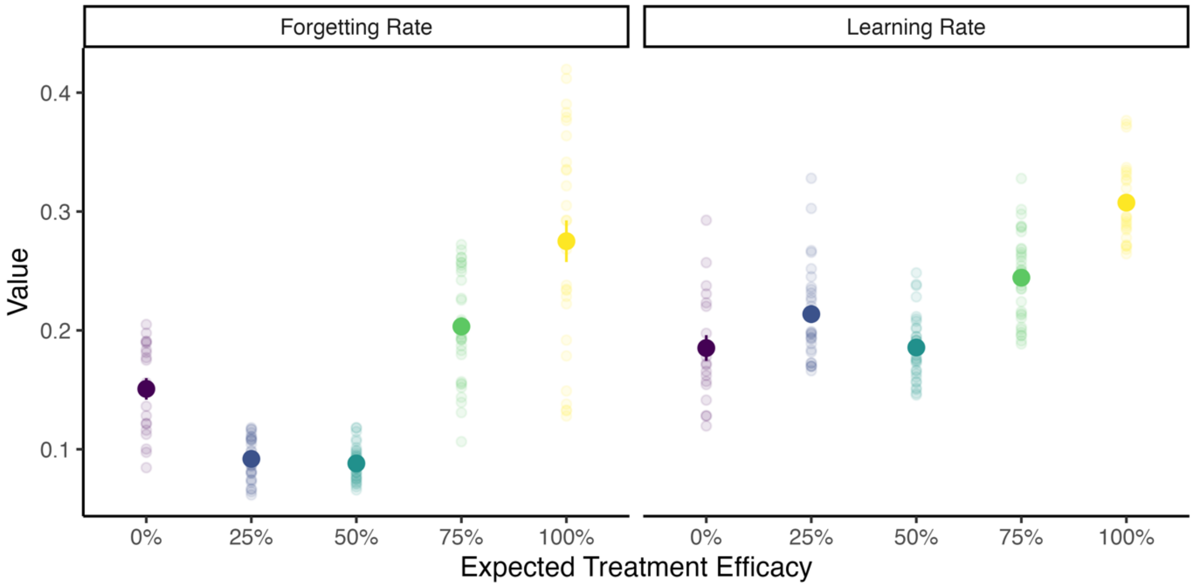

Supplementary Figure S7. Individual posterior means for forgetting and learning rates obtained for the RLf-CK model, separately for each treatment group. Treatment groups are represented on the x-axis, (constrained) individual parameter posterior means on the y-axis. Bold dots indicate the group-level mean, and error bars represent the standard error of the mean.
